# Reliability of Manual Measurements Versus Semiautomated Software for Glenoid Bone Loss Quantification in Patients With Anterior Shoulder Instability

**DOI:** 10.1177/23259671231222938

**Published:** 2024-02-12

**Authors:** Katrin Karpinski, Doruk Akguen, Henry Gebauer, Alp Paksoy, Mattia Lupetti, Viktoria Markova, Oliver Zettinig, Philipp Moroder

**Affiliations:** * Centrum für Muskuloskeletale Chirurgie, Charité–Universitätsmedizin Berlin, Berlin, Germany; † ImFusion, Munich, Germany; ‡ Schulthess Klinik, Zürich, Switzerland; Investigation performed at Centrum für Muskuloskeletale Chirurgie, Charité–Universitätsmedizin Berlin, Berlin, Germany

**Keywords:** shoulder instability, glenoid bone loss, glenoid defect, BSSR, glenoid concavity, shoulder segmentation software

## Abstract

**Background:**

The presence of glenoid bone defects is indicative in the choice of treatment for patients with anterior shoulder instability. In contrast to traditional linear- and area-based measurements, techniques such as the consideration of glenoid concavity have been proposed and validated.

**Purpose:**

To compare the reliability of linear (1-dimensional [1D]), area (2-dimensional [2D]), and concavity (3-dimensional [3D]) measurements to quantify glenoid bone loss performed manually and to analyze how automated measurements affect reliability.

**Study Design:**

Cohort study (diagnosis); Level of evidence, 3.

**Methods:**

Computed tomography images of 100 patients treated for anterior shoulder instability with differently sized glenoid defects were evaluated independently by 2 orthopaedic surgeons manually using conventional software (OsiriX; Pixmeo) as well as automatically with a dedicated prototype software program (ImFusion Suite; ImFusion). Parameters obtained included 1D (defect diameter, best-fit circle diameter), 2D (defect area, best-fit circle area), and 3D (bony shoulder stability ratio) measurements. Mean values and reliability as expressed by the intraclass correlation coefficient [ICC]) were compared between the manual and automated measurements.

**Results:**

When manually obtained, the measurements showed almost perfect agreement for 1D parameters (ICC = 0.83), substantial agreement for 2D parameters (ICC = 0.79), and moderate agreement for the 3D parameter (ICC = 0.48). When measurements were aided by automated software, the agreement between raters was almost perfect for all parameters (ICC = 0.90 for 1D, 2D, and 3D). There was a significant difference in mean values between manually versus automatically obtained measurements for 1D, 2D, and 3D parameters (*P* < .001 for all).

**Conclusion:**

While more advanced measurement techniques that take glenoid concavity into account are more accurate in determining the biomechanical relevance of glenoid bone loss, our study showed that the reliability of manually performed, more complex measurements was moderate.

The prevalence of glenoid bone loss is as high as 90% in patients with recurrent anterior shoulder instability.^24^ An increasing number of glenoid bony defects has been shown to have a negative impact on shoulder stability in biomechanical studies.^29,30^ The appropriate treatment for patients suffering from anterior shoulder instability is mainly dependent on the extent of glenoid bone loss,^8^ as it negatively affects the success of standard soft tissue stabilization procedures.^5,7,25^ Consequently, different values of “critical” glenoid bone loss have been reported in the literature that guide treating physicians toward performing bony reconstruction surgery to restore the normal glenoid shape.^16,23,30^

Several different measurement techniques are currently available to measure glenoid bone loss; however, a universally accepted method does not exist, and surgical decision making regarding glenoid deficiency is subjective.^4,14,26^ Proposed measurement methods for the quantification of glenoid bone loss include linear-based (1-dimensional [1D]) and surface area–based (2-dimensional [2D]) techniques in the en face view of 3-dimensional (3D) computed tomography (CT), which is preferred over 2D CT and magnetic resonance imaging (MRI).^20,26^ However, these techniques do not consider the native glenoid concavity, which was recently shown to have a higher impact on shoulder stability than the size of a bony defect.^3,17,27,28^ These concavity-based (3D) measurements are not only able to predict the biomechanical effect of various degrees of glenoid bone loss more accurately,^16^ but they can also be used to consider glenoid concavity differences between patients that alter the effect of glenoid bone loss.^16,17^ The calculation of glenoid bone loss presents different results depending on the measurement technique with varying reliability and accuracy values.^1^ Concern exists that with increasing complexity of the technique used to measure glenoid bone loss, manual performance of these measurements might not be sufficiently reliable.

The aim of this study was to compare the reliability of 1D (linear), 2D (area), and 3D (concavity) quantification techniques of glenoid bone loss performed manually using conventional imaging software and to analyze how automated measurements with dedicated software affect reliability. The hypothesis of this study was that software that automatically analyzes glenoid bony anatomy would be more reliable in determining glenoid bone loss than manual measurements, especially regarding more complex parameters.

## Methods

### Patient Cohort

The CT images of 100 shoulders in 100 consecutive patients treated in our clinic between January 2018 and December 2020 with anterior shoulder instability and anterior glenoid bone loss (regardless of the extent) were collected; the CT scan slice thickness was 0.625 mm. We excluded patients with previous shoulder stabilization surgery. Of the cohort, 56 patients received arthroscopic Bankart or Bankart-plus repair,^15^ 36 were treated by J-bone grafting, and 8 underwent the Latarjet procedure. There were 84 male and 16 female patients (47 left and 53 right shoulders) with a mean age of 31.2 ± 7.5 years (range, 18-55 years).

### Manual Measurements

Digital Imaging and Communications in Medicine (DICOM) data of the patients’ shoulder CT images (n = 100) were rendered into 3D models using the image-processing software OsiriX (Pixmeo). The en face view of the glenoid was selected, and the best-fit circle (BFC) was placed on the inferior aspect of the remaining glenoid rim^24^ manually by 2 experienced shoulder surgeons using the spoon technique, as previously described.^18^

**Figure 1.**
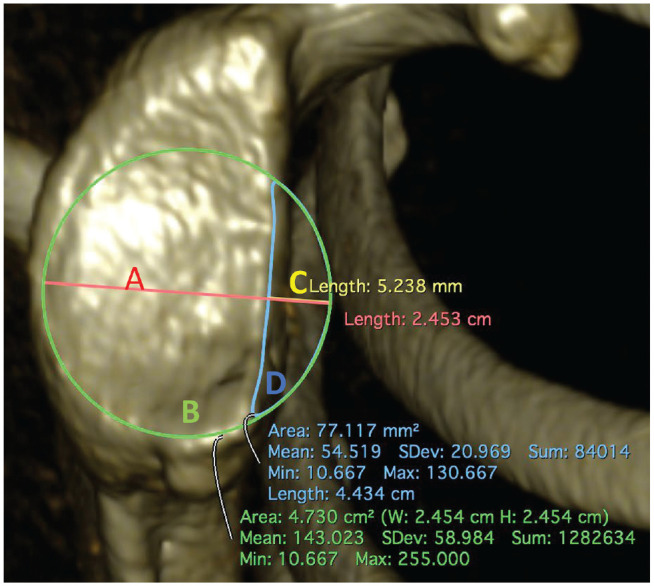
En face view of the glenoid on a 3-dimensional CT. The diameter (*A*; red) and the area (*B*; green) of the BFC positioned on the inferior aspect of the glenoid were determined. The extent of the defect was determined 1-dimensionally by assessing the defect diameter (*C*; yellow) in relation to the BFC diameter (*A*) and 2-dimensionally by assessing the defect area (*D*; blue) in relation to the BFC area (*B*).

The diameter and area of the BFC were determined for each patient. Linear measurements (1D) were performed as follows. The glenoid defect diameter, defined as the longest perpendicular distance between the anterior glenoid rim and the BFC, was measured (Figure 1). The ratio between the defect diameter and the diameter of the BFC constituted relative glenoid bone loss. The PICO method was used to determine area-based (2D) glenoid bone loss.^2^ Therefore, the area of the glenoid defect was measured according to Baudi et al.^2^ Relative glenoid bone loss was calculated as the ratio between the area of the defect and the area of the BFC (Figure 1).

**Figure 2.**
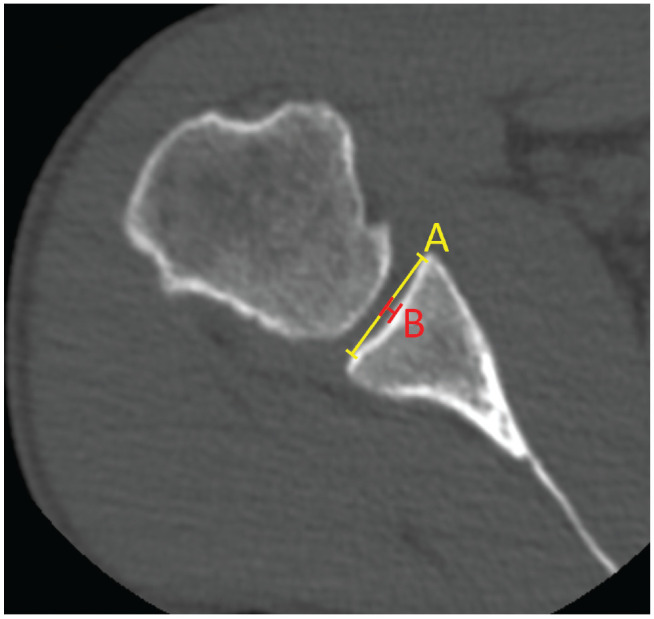
For calculating the BSSR, the concavity diameter obtained by drawing a tangent line from one apex of the concavity to the opposite concavity (*A*; yellow line) as well as the concavity depth, defined as the distance from the deepest point of the concavity to the tangent line (*B*; red line), were determined, and the BSSR was calculated according to Moroder et al.^17^ Multiplanar reconstruction was used to obtain standardized axial images that were perpendicular to the long axis of the glenoid and passed through the center of the best-fit circle.

For calculating the 3D bony shoulder stability ratio (BSSR),^17^ the concavity diameter as well as the concavity depth were determined. Multiplanar reconstruction was used to obtain standardized axial images that were perpendicular to the long axis of the glenoid and passed through the center of the BFC.^19^ The concavity diameter was then obtained by drawing a tangent line from one apex of the concavity to the opposite concavity. The distance from the deepest point of the concavity to the tangent line constituted the depth (Figure 2). The BSSR was consequently calculated according to the method of Moroder et al.^17^

### Automatic Measurements

**Figure 3.**
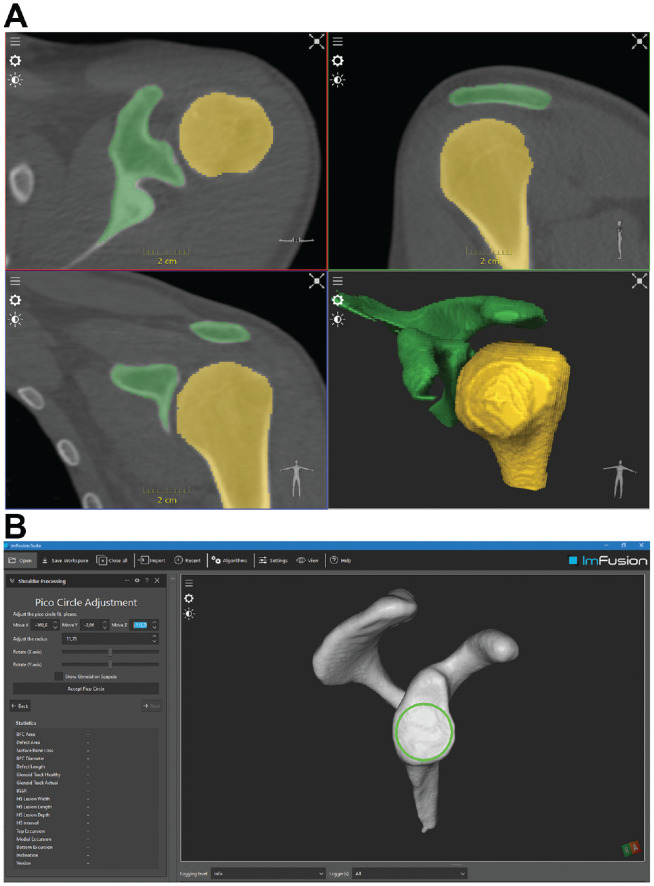
Computed tomography–based measurements conducted automatically using ImFusion software. (A) The result was a label map representing the scapula and the humerus. The segmentation output of the humerus and scapula was reviewed and refined by the user. (B) Consequently, the geometric properties of the glenoid were characterized and the best-fit circle estimated for further refinement.

For computer-assisted measurements, a dedicated software program (ImFusion Suite; ImFusion) was employed. The software workflow consisted of several steps. The DICOM files of the CT image were first loaded and interpreted as 3D volumetric data. A deep learning (DL) model was then run on the data to create a segmentation map describing the humerus and the scapula (Figure 3A).

The DL model was prepared using the open-source framework TensorFlow (https://www.tensorflow.org), which output a pixelwise classification of the input image. The result was a label map representing the scapula and the humerus. The DL model’s segmentation output was reviewed and refined by each user, with particular attention to the region of the glenohumeral joint, which was critical for all successive analysis steps (Figure 3A). Once the segmentations were reviewed and accepted, the geometric properties of the glenoid were characterized. Therefore, surface meshes were extracted from the volumetric segmentations to outline the glenoid as a subregion of the scapular mesh. This was then visually refined by the user.

After the glenoid mesh was accepted, its geometry was characterized by computing its long, short, and normal axes by means of principal component analysis of the mesh vertices. The long axis pointed toward the patient’s head, the short axis toward the patient’s front side, and the normal axis toward the humeral head. The vertices’ centroid was then used as the origin of the reference frame generated by the principal axes. Given the glenoid mesh and its reference frame, the BFC was estimated as follows. The glenoid mesh was split into 4 quadrants identified by its long and short axes, and only the posteroinferior quadrant was considered. This amounted to considering only the lower rear quarter of the glenoid mesh. Of this mesh portion, the vertices closest to the mesh border were used for best fitting a circle using the previously mentioned spoon technique, which was then presented to the user as the BFC estimate and could be further manually refined (Figure 3B).

**Figure 4.**
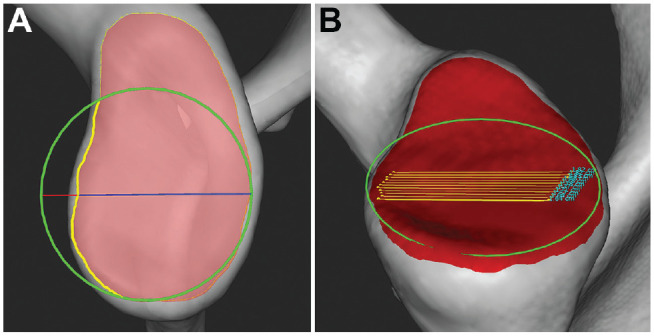
Evaluation of a shoulder CT image using ImFusion software. (A) Linear- and area-based glenoid bone loss were calculated by projecting the glenoid mesh contour (red shaded area) onto the BFC plane (green circle). Linear-based glenoid bone loss was defined as the maximum perpendicular distance between the BFC and the glenoid contour projection (red line), and area-based glenoid bone loss was defined as the BFC area minus the area of the glenoid contour projection inside the BFC (yellow line). (B) To compute the BSSR, the concavity depth was determined. For this purpose, the glenoid mesh was cut along its short axis by a plane passing through the BFC center, and the concavity depth was estimated as the farthest point from a line parallel to the glenoid short axis that touched the glenoid mesh. As the measurement is very sensitive to glenoid mesh irregularities, the calculation was repeated along 10 different mesh cuts, performed with planes parallel to the first plane (yellow lines), to produce the final concavity depth value as an average.

As soon as the BFC was reviewed, glenoid bone loss and the BSSR were quantified (Figure 4). Both linear- and area-based glenoid bone loss were estimated by projecting the glenoid mesh contour onto the BFC plane (Figure 4A). Linear-based (1D) glenoid bone loss was calculated as the ratio between the defect diameter, defined as the maximum distance between the BFC and the glenoid contour projection, and the diameter of the BFC. Area-based (2D) glenoid bone loss was calculated as the ratio between the area of the defect, defined as the BFC area minus the area of the glenoid contour projection inside the BFC, and the area of the BFC. To compute the BSSR, the concavity depth was determined. For this purpose, the glenoid mesh was cut along its short axis by a plane passing through the BFC center. On the resulting intersection points, the concavity depth was estimated as the farthest point from a line parallel to the glenoid short axis that touched the glenoid mesh without intersecting it (Figure 4B). As the measurement is very sensitive to glenoid mesh irregularities, the calculation was repeated along 10 different mesh cuts, performed with planes parallel to the plane described above, to produce the final concavity depth value as an average. The measurements collected in the software workflow were then saved to a report for further analysis.^17^

### Statistical Analysis

The measurements were independently conducted by 2 orthopaedic fellows (K.K. and D.A.), who were both blinded to the patients’ charts. Statistical analysis was performed using SPSS Statistics software (Version 27.0; IBM). Intraclass correlation coefficients (ICCs) were calculated with 95% confidence intervals to determine the interobserver reliability for all measurements. ICC values were interpreted according to Landis and Koch^10^ in which ≤0.20 was considered slight agreement, 0.21 to 0.40 indicated fair agreement, 0.41 to 0.60 indicated moderate agreement, 0.61 to 0.80 indicated substantial agreement, and ≥0.81 indicated almost perfect agreement. After the reliability assessment, the values of both raters were averaged for further analysis. Furthermore, the mean values of every parameter for both raters were calculated including the standard deviation and range. The parameters were tested for normal distribution using the Kolmogorov-Smirnov test. To compare manually and automatically obtained measurements, the paired *t*-test was used for normally distributed data, and the Wilcoxon test was used for nonnormally distributed data. *P* < .05 was considered to be statistically significant.

## Results

Measurement results for all parameters are summarized in Table 1. There was a significant difference between all manually and automatically obtained measurements, with manually performed 1D and 2D measurements rendering lower values for the extent of the glenoid defect compared to automated measurements. The percentage of glenoid bone loss was 12% ± 7% for 1D manual measurements and 17% ± 10% for 1D automated measurements (*P* < .001) as well as 8% ± 5% for 2D manual measurements and 13% ± 9% for 2D automated measurements (*P* < .001). The 3D BSSR was significantly higher with manual measurements compared to automated measurements (20% ± 9% vs 15% ± 6%, respectively; *P* < .001).

**Table 1:** Comparison of Values by Manual Versus Automated Measurements^*a*^

	Manual Measurements (OsiriX Software; n = 100)	Automated Measurements (ImFusion Software; n = 100)	*P*
Linear (1-dimensional)			
BFC diameter, mm	29 ± 3 (22-35)	25 ± 3 (18-32)	**<.001**
Defect diameter, mm	4 ± 2 (0-10)	4 ± 3 (0-12)	**<.001**
Ratio, %	12 ± 7 (0-32)	17 ± 10 (0-47)	**<.001**
Area (2-dimensional)			
BFC area, mm^2^	645 ± 113 (363-940)	485 ± 95 (259-786)	**<.001**
Defect area, mm^2^	54 ± 41 (0-250)	64 ± 50 (0-254)	**<.001**
Ratio, %	8 ± 5 (0-30)	13 ± 9 (0-56)	**<.001**
Concavity (3-dimensional)			
BSSR, %	20 ± 9 (3-43)	15 ± 6 (3-31)	**<.001**

a Data are reported as mean ± SD (range). Boldface *P* values indicate a statistically significant difference between groups (*P* < .05). BFC, best-fit circle; BSSR, bony shoulder stability ratio.

The interrater reliability of the manual measurements was found to be almost perfect for 1D parameters (ICC = 0.83), substantial for 2D parameters (ICC = 0.79), and moderate for the 3D parameter (ICC = 0.48). The interrater reliability of the automated measurements was found to be almost perfect for all of the 1D, 2D, and 3D parameters (ICC = 0.90 for all) (Table 2).

**Table 2:** Interrater Reliability of Manual and Automated Measurements^*a*^

	Manual Measurements		Automated Measurements	
	ICC (95% CI)	Agreement	ICC (95% CI)	Agreement
Linear (1-dimensional)				
BFC diameter	0.8 (0.5-0.9)	Substantial	0.9 (0.7-1.0)	Almost perfect
Defect diameter	0.8 (0.7-0.9)	Substantial	0.9 (0.9-1.0)	Almost perfect
Ratio	0.8 (0.7-0.9)	Almost perfect	0.9 (0.9-1.0)	Almost perfect
Area (2-dimensional)				
BFC area	0.8 (0.4-0.9)	Substantial	0.9 (0.7-1.0)	Almost perfect
Defect area	0.8 (0.6-0.8)	Substantial	0.9 (0.9-1.0)	Almost perfect
Ratio	0.8 (0.7-0.9)	Substantial	0.9 (0.8-0.9)	Almost perfect
Concavity (3-dimensional)				
BSSR	0.5 (0.2-0.7)	Moderate	0.9 (0.9-0.9)	Almost perfect

a BFC, best-fit circle; BSSR, bony shoulder stability ratio; ICC, intraclass correlation coefficient.

## Discussion

The most important finding of the study was that more complex measurements seemed to be more reliable when performed by dedicated software. After comparing the reliability of 1D, 2D, and 3D glenoid bone loss quantification techniques, we found, as expected, that reliability decreased as the complexity of the manually performed measurement technique increased. Thus, the interrater reliability of manual measurements was found to be almost perfect for 1D parameters, substantial for 2D parameters, and moderate for the 3D parameter. However, this was not true for automated measurements performed by software, with almost perfect agreement for all parameters. The possible reasons for an error in manual measurements can be the lack of standardization of en face view orientation and BFC placement. Moroder et al^18^ showed a significant alteration in measurement results of the glenoid defect size caused by imprecision of scapular positioning in the en face view of the glenoid as well as varying the BFC placement. The overall agreement regarding en face view image selection between the observers was only 30%.^18^ Furthermore, manual measurements of the 3D shape of the glenoid, including concavity, represent a challenge and can be performed more precisely by automated software, as shown in our study.

The lack of a universally accepted preoperative method to quantify glenoid bone loss, the low reliability of currently established measurement methods, and subjective decision-making regarding glenoid deficiency are possibly reasons for progressive changes in critical bone loss values over time in the literature, reaching as low as 13%.^13,21,22^ Following this, Chalmers et al^6^ showed that differences in measurement methods with the lack of a gold standard may lead to differences in the choice of treatment in up to 34% of cases, with the most aggressive treatment recommendations associated with linear-based (1D) CT measurements. This seems to be mostly true, especially for manually performed measurements, as manually performed 1D and 2D measurements tend to underestimate the glenoid defect compared to automated measurements, as shown in our study possibly because of the above mentioned errors. Moreover, the recent literature questions the sufficiency of 1D and 2D measurements in not taking into account the native glenoid concavity, which seems to play a crucial role in terms of the concavity-compression effect. Several studies have described the biomechanical relationship between the 3D shape of the glenoid and the stability ratio.^9,11,12,17,27^ Furthermore, Moroder et al^16,17^ emphasized the differences in glenoid morphology between patients and challenged the current concept of defining a general threshold for a critical glenoid defect. In their finite element analysis, they showed a nonlinear relationship between the glenoid defect size and its biomechanical effect and differences in biomechanically relevant glenoid concavity between patients. Based on these findings, small glenoid defects might have a higher impact on stability than previously recognized, and generally, the biomechanical effect of glenoid bone loss depends on glenoid concavity, which varies among patients. These findings were confirmed in a biomechanical study by Wermers et al,^28^ who showed that the stability ratio was significantly dependent on glenoid concavity, whereas the defect size had only a minor influence, concluding that glenoid concavity has the potential to significantly influence clinical decision-making for improved and personalized treatment of glenohumeral instability. While the best available technique to measure glenoid bone loss is still debated, 3D measurements of glenoid bone loss seem to be the most accurate method to determine the biomechanical effect of glenoid bone loss on glenohumeral stability. As manual measurements of glenoid concavity have limited reliability, it is of importance that automated measurement software should be used in the clinical setting to improve reliability.

### Limitations

This study has some limitations, including the lack of analyzing subgroups in terms of traumatic versus atraumatic and recurrent versus primary instability to examine different rim shapes and perform a breakdown of measuring defect sizes with regard to critical bone loss. Furthermore, in this study, only the ICC was calculated. Another limitation of the software is that it still requires manual adjustments in some cases, which can affect reliability. However, this necessity is likely to be reduced by improving the automated steps via DL modeling. A limitation of this study is that all measurements, both manually or automatically performed, relied on the quality of CT, which varied depending on where and how CT had been performed. However, this would equally affect both types of measurement techniques. Finally, this study only provides answers regarding the reliability of automated measurements and does not represent a validation of their accuracy, which yet needs to be proven. Further studies will be needed to propose a method, taking into account the concavity shape and rim defect together, to arrive at a precise decision regarding therapy.

## Conclusion

While it has been shown that more advanced measurement techniques that take glenoid concavity into account are more accurate in determining the biomechanical relevance of glenoid bone loss, our study showed that the reliability of manually performed, more complex measurement techniques was moderate. Automated measurements using dedicated computer software may improve the reliability for all types of measurement techniques to an almost perfect level, and therefore, its use should be considered for future research and clinical use. This could create a more standardized platform to assess the extent of glenoid defects with potential impacts on future clinical decision-making.
